# Plastic and Neuroprotective Mechanisms Involved in the Therapeutic Effects of Cannabidiol in Psychiatric Disorders

**DOI:** 10.3389/fphar.2017.00269

**Published:** 2017-05-23

**Authors:** Alline C. Campos, Manoela V. Fogaça, Franciele F. Scarante, Sâmia R. L. Joca, Amanda J. Sales, Felipe V. Gomes, Andreza B. Sonego, Naielly S. Rodrigues, Ismael Galve-Roperh, Francisco S. Guimarães

**Affiliations:** ^1^Department of Pharmacology, Centre for Interdisciplinary Research on Applied Neurosciences (NAPNA), School of Medicine of Ribeirão Preto, University of São PauloRibeirão Preto, Brazil; ^2^Department of Physical and Chemical, School of Pharmaceutical Science of Ribeirão Preto, University of São PauloRibeirão Preto, Brazil; ^3^Department of Neuroscience, University of PittsburghPittsburgh, PA, United States; ^4^Department of Biochemistry and Molecular Biology I, School of Biology, Complutense UniversityMadrid, Spain; ^5^Centro de Investigación Biomédica en Red sobre Enfermedades Neurodegenerativas, Instituto de Universitario de Investigación en Neuroquímica and Instituto Ramón y Cajal de Investigación SanitariaMadrid, Spain

**Keywords:** cannabinoids, anxiety, depression, schizophrenia, neurogenesis, synaptic remodeling, autophagy

## Abstract

Beneficial effects of cannabidiol (CBD) have been described for a wide range of psychiatric disorders, including anxiety, psychosis, and depression. The mechanisms responsible for these effects, however, are still poorly understood. Similar to clinical antidepressant or atypical antipsychotic drugs, recent findings clearly indicate that CBD, either acutely or repeatedly administered, induces plastic changes. For example, CBD attenuates the decrease in hippocampal neurogenesis and dendrite spines density induced by chronic stress and prevents microglia activation and the decrease in the number of parvalbumin-positive GABA neurons in a pharmacological model of schizophrenia. More recently, it was found that CBD modulates cell fate regulatory pathways such as autophagy and others critical pathways for neuronal survival in neurodegenerative experimental models, suggesting the potential benefit of CBD treatment for psychiatric/cognitive symptoms associated with neurodegeneration. These changes and their possible association with CBD beneficial effects in psychiatric disorders are reviewed here.

## Introduction

Plasticity relates to the particular characteristic of a material that undergoes deformation under a load (Lubliner, 2005). In neuroscience, the term neuroplasticity applies to the capacity of the brain to adapt and change in response to experience (Fuchs and Flugge, 2014). William James was the first to propose this term in 1890, defending the idea that brain functions are not fixed during life (James, 1890). Several neuroscientists denied this concept for decades. Santiago Ramón y Cajal, however, used the term neuroplasticity to describe changes in the brain that were a consequence of, or related to, pathology. He also suggested that small protrusions in the dendrites of neurons stained with Golgi's method, which he later named as dendritic spines, are involved in synaptic connectivity and function (Stahnisch and Nitsch, 2002). Nowadays, the idea that brain continually changes along our lifetime is well accepted. Notably, the concept of neuroplasticity has expanded to include not only changes at a morphological level but also biochemical and pharmacological adaptations (intracellular pathways, receptors, synaptic proteins), alterations in neuronal networks (changes in connectivity, dendritic remodeling, and number and morphology of dendritic spines), as well as the generation of new neurons (i.e., adult neurogenesis) (Fuchs and Flugge, 2014). These neuroplastic modifications, moreover, can be either adaptive or maladaptive. Therefore, the mechanisms responsible for these changes may be a great window of opportunity for understanding the pathophysiology and treatment of mental illness (Kays et al., 2012).

### Neuroplasticity and psychotropic drugs

Psychiatric disorders may result from significant neuroplastic changes that lead to new set points of brain functions (Pallanti, 2016). For instance, several neuropsychiatric conditions have been associated with stress-induced changes in dendritic remodeling and decreased adult hippocampal neurogenesis (Bessa et al., 2009; Campos et al., 2013b). Corroborating this proposal, decreased hippocampal volume and reduced proliferative activity of neurogenic niches have been described in mood disorders, posttraumatic stress disorder (PTSD) and schizophrenia (Reif et al., 2006; Dhikav and Anand, 2007; Lucassen et al., 2010).

The therapeutic effects of several psychotropic drugs usually need 2–6 weeks to be clinically recognized. It suggests that time-dependent structural reorganization of neuronal circuits and biochemical synaptic changes are required for the pharmacological action of these drugs (Konradi and Heckers, 2001; Fogaça et al., 2013).

Antidepressants are probably the most studied class of medication associated with plastic brain changes. For example, chronic, but not acute, treatment with antidepressants such as serotonin selective uptake inhibitors (SSRIs) and tricyclics increases the expression of Brain-derived Neurotrophic Factor (BDNF) in the hippocampus and prefrontal cortex (PFC) (Castren et al., 2007). Repeated antidepressant treatment also prevents stress-induced hippocampal dendritic atrophy (Bessa et al., 2009) and facilitates adult hippocampal neurogenesis in rodents (Malberg et al., 2000; Santarelli et al., 2003). In addition to standard antidepressant drugs, the rapid and sustained antidepressant effects induced by ketamine also seem to depend on neuroplastic events (Duman et al., 2016). Ketamine appears to act in the PFC and hippocampus modifying the number of dendritic spines and BDNF expression by facilitating mTOR (mechanistic Target of Rapamycin) intracellular pathway (Duman et al., 2016).

Neuroplastic changes have also been associated with the effects of antipsychotic drugs. Haloperidol modifies the number/shape of dendritic spines and synaptic strength and increases expression of synaptic proteins (Eastwood et al., 1997; Harris, 1999; Matus, 1999; Nakahara et al., 1999). Regarding adult hippocampal neurogenesis, the results are contradictory. Whereas Malberg et al. (2000) found no changes in haloperidol-treated adult rats, in gerbils haloperidol seems to facilitate neurogenesis (Dawirs et al., 1998). In the case of atypical antipsychotic drugs, clozapine induces proliferation in the subgranular zone of the rodent dentate gyrus 24-h after the treatment (Halim et al., 2004) and prevents the phencyclidine-induced decrease in hippocampal neurogenesis (Maeda et al., 2007).

### Cannabidiol effects in psychiatric disorders

Cannabidiol (CBD) is one of the most abundant components among more than 100 compounds called cannabinoids present in the *Cannabis sativa* plant. CBD differs from it's the main psychoactive component, delta-9-tetrahydrocannabinol (THC), CBD does not cause psychotomimetic and anxiogenic effects or induce dependence after repeated use (for review, see Ligresti et al., 2016). In addition, it has a better safety profile compared to other cannabinoids, such as THC. For instance, high doses of CBD (up to 1,500 mg/day) are well tolerated in animals and humans.

Nowadays, CBD is one of the phytocannabinoid with the widest range of potential therapeutic actions (Izzo et al., 2009; Ligresti et al., 2016). There are a considerable number of clinical trials using CBD alone or in combination with other cannabinoids in progress (Campos et al., 2016). CBD has attracted considerable interest recently, as marihuana extracts enriched in CBD have been reported to exert a significant reduction in seizure number and severity in Dravet and Gaston-Leroux patients (Devinsky et al., 2014). Of note, the Food and Drug Administration and, the European Medicines Agency approved the use of CBD (Epidiolex, GW) for the treatment of this conditions. Additionaly, CBD exhibits a broad spectrum of other possible therapeutic actions, which include anxiolytic, antipsychotic, antidepressive, and neuroprotective effects over a large range of psychiatric and neurodegenerative disorders (Campos et al., 2016; Ligresti et al., 2016). Although most of these putative therapeutic properties were initially described in animal models, clinical studies have supported the beneficial effects of CBD in anxiety, schizophrenia, epilepsy, and multiple sclerosis (Bergamaschi et al., 2011a; Leweke et al., 2012; Ligresti et al., 2016; Table 1). Corroborating these findings, neuroimaging studies clearly demonstrated that CBD affects brain areas involved in the neurobiology of psychiatric disorders. Crippa et al. (2004) showed that a single dose of CBD, administered orally in healthy volunteers, alters the resting activity in limbic and paralimbic brain areas while decreasing subjective anxiety associated with the scanning procedure. CBD reduced the activity of the left amygdala-hippocampal complex, hypothalamus, and posterior cingulated cortex while increasing the activity of the left parahippocampal gyrus compared with placebo. In healthy volunteers treated with CBD and submitted to a presentation of fearful faces, a decreasing of the amygdala and anterior and posterior cingulate cortex activities and a disruption in the amygdala-anterior cingulated cortex connectivity have also been observed (Fusar-Poli et al., 2009, 2010). Furhter imaging studies also demonstrated that CBD changes activity in other brain areas involved in neuropsychiatric disorders such as the medial and left temporal and prefrontal cortex and insula (Borgwardt et al., 2008; Bhattacharyya et al., 2010; Table 1).

**Table 1:** **CBD effects in psychiatric disorders**.

**Behavioral effect**	**Model**	**CBD dose/concentration range**	**Route of administration/schedule**	**Species/Strain**	**Mechanisms investigated**	**References**
**PRECLINICAL STUDIES**						
Antidepressant-like	Forced Swimming Test (FST)	30 mg/kg	Acute, i.p.	Swiss mice	5HT_1A_	Zanelati et al., 2010
	FST and Tail Suspension Test (TST)	200 mg/kg	Acute, i.p.	Swiss Webster mice (FST) DBA/2 mice (TST)	Not determinated	El-Alfya et al., 2010
	FST	30 mg/kg	Acute and chronic, i.p.	Wistar rats	–	Réus et al., 2011
Antidepressant-/Anxiolytic-like	Chronic Unpredictable Stress	30 mg/kg	Chronic, i.p.	GFAP-thymidine kinase (GFAP-TK) transgenic mice	CB1, increased neurogenesis and anandamide levels	Campos et al., 2013b
	Novelty Suppressed Feeding			C57BL6 mice		
	Elevated Plus Maze (EPM)					
Antidepressant-like	FST and TST	3 and 30 mg/kg	Acute and chronic, i.p.	Swiss mice	Increased neurogenesis	Schiavon et al., 2016
	Olfactory bulbectomy	50 mg/kg	Acute and chronic, i.p.	C57BL6 mice	5HT_1A_	Linge et al., 2016
	FST		Intracerebral (mPFC), acute	Wistar rats	5HT_1A_	Sartim et al., 2016
	FST	30 mg/kg	Acute, i.p.	Swiss mice	Not determinated	Breuer et al., 2016
	Saccharin consumption test	30 mg/kg	Oral, acute	Wistar-Kyoto (WKY) rat	Not determinated	Shoval et al., 2016
Antipsychotic	Repeated administration of the NMDA receptor antagonist MK-801	15–60 mg/kg	14 days, i.p.	C57BL6/J mice	Attenuated parvalbumin loss and glial activation in the mPFC,	Gomes et al., 2015a,b
	Amphetamine sensitization model Prepulse inhibition (PPI)	100 ng/0.5 μL	Intra-NAc shell/acute	Sprague Dawley rats	Attenuated PPI disruption and increased dopamine system activity via a mTOR/p70S6Kinase signaling pathway	Renard et al., 2016
	Acute administration of the NMDA receptor antagonist MK-801	5 mg/kg	Acute, i.p.	Swiss mice	TRPV1 receptors	Long et al., 2006
Anxiolytic-like	EPM Vogel‘s conflict test	30 nmol	Intra-periqueductal gray matter	Wistar rats	5HT_1A_	Campos and Guimarães, 2008
	EPM	60 nmol	Intra-periqueductal gray matter	Wistar rats	TRPV1	Campos and Guimarães, 2009
	Predator threat-induced long lasting behavioral alterations	5 mg/kg	7 days, i.p.	Wistar rats	5HT_1A_	Campos et al., 2012a
	Elevated T-Maze	5 mg/kg	21 days, i.p.	Wistar rats	5HT_1A_	Campos et al., 2013a
	Marble burying	15–60 mg/kg	Acute, i.p.	Swiss mice	CB1	Casarotto et al., 2010
Anxiogenic-like	Contextual Fear conditioning	10 mg/kg	14 days, i.p.	Lister-hooded rats	Decreased levels of the phosphorylated form of ERK1/2 in the PFC	ElBatsh et al., 2012
**CLINICAL STUDIES**						
Antipsychotic	Double blind controlled clinical trial	600–800 mg	28 days, oral	Schizophrenia patients	Reduces psychotic symptoms similar to amisulpride	Leweke et al., 2012
	Placebo-controlled clinical trial	Not informed	Oral	Schizophrenia patients	Reduces psychotic symptoms in patients that have previously failed to respond adequately to first line anti-psychotic medications	GW Pharmaceuticals, 2015
Anxiolytic		400 mg	Acute, oral		↓ Subjective anxiety and ↑ mental sedation.	Crippa et al., 2004
					↓ Blood Flow in posterior cingulated cortex and Amygdala/Bed nucleus of stria terminalis and ↑ in left parahippocampal gyrus	
		600 mg	Acute, oral		↓ Blood-oxygen-level dependent contrast imaging (BOLD) of amydala signal and amygdala-anterior cingulated connectivity during fearful faces presentations	Fusar-Poli et al., 2009, 2010
		600 mg	Acute, oral		↓ Activation left temporal and insular cortex during motor inhibition task	Borgwardt et al., 2008

*i.p., intraperitoneal; mPFC, medial prefrontal córtex; ↓, decreases; ↑, increases*.

## Mechanisms of CBD effects in neuropsychiatric disorders

The mechanism of action of CBD remains controversial and is yet unclear. Numerous signaling pathways have been proposed as candidates to mediate its neuroplastic effects (Izzo et al., 2009; Fernández-Ruiz et al., 2013). The possible targets of CBD action have been extensively examined recently (Ibeas Bih et al., 2015; Ligresti et al., 2016). The present review will focus on mechanisms more closely associated with the neuropsychiatric effects of CBD, particularly those related to its neuroplastic effects.

Although several CBD actions are not directly mediated by canonical metabotropic CB_1_/CB_2_ receptors, CBD can influence endocannabinoid (ECB) levels (Izzo et al., 2009). While there are contradictory results, it was suggested that CBD exerts an antagonist or negative modulatory action, but with a low affinity, at CB_1_ and CB_2_ receptors (Thomas et al., 2007; Laprairie et al., 2015; McPartland et al., 2015). At high concentrations, CBD can facilitate ECB-mediated actions *in vitro*, including CB_1_-neuromodulation by decreasing their hydrolysis mediated by Fatty acid amide hydrolase (FAAH) and Monoacylglycerol lipase (MAGL) or re-uptake (Bisogno et al., 2001; De Petrocellis et al., 2011). Another possible mechanism by which CBD decreases anandamide uptake/metabolism in humans, but not rodents, could be the binding to fatty acid binding proteins (FABPs), which is necessary for the transport of this ECB from the membrane to intracellular FAAH (Elmes et al., 2015). The interaction of plant-derived cannabinoids with the ECB metabolism enzymes explains partially why some of the anxiolytic effects of CBD are mediated by CB_1_ receptors (Casarotto et al., 2010; Campos et al., 2013a).

Like other cannabinoids, CBD produces bell-shaped dose-response curves and can act by different mechanisms accordingly to its concentration or the simultaneous presence of other cannabinoid-ligands (Campos et al., 2012b; Ligresti et al., 2016). CBD can regulate, directly or indirectly, the activity of peroxisome proliferator-activated receptor gamma (PPARγ), serotonin 5HT_1A_ receptor, adenosine transporter, members of the TRPV family, and the metabotropic CB_1_ and CB_2_ receptors (Campos et al., 2012b; Ligresti et al., 2016).

### 5HT_1A_ receptors and the effects of CBD

The acute anxiolytic and antidepressive actions of acute CBD are proposed to be mediated by serotonin 5HT_1A_ receptors. The crosstalk between cannabinoids and serotoninergic signaling, however, is complex. In rats, CBD administration into the dorsal portions of periaqueductal gray matter (dPAG) produces anti-aversive effects in the elevated plus maze and flight-induced by local electric stimulation. These effects were prevented by WAY-100635, a 5HT_1A_ antagonist (Campos and Guimarães, 2008; Soares et al., 2010). Other brain regions, such as the basal ganglia (Espejo-Porras et al., 2013), the bed nucleus of stria terminallis (Gomes et al., 2011), the prelimbic PFC (Fogaça et al., 2014) and the dorsal raphe nucleus (Rock et al., 2012; Katsidoni et al., 2013), also seem to mediate CBD effects via 5HT_1A_ receptors. Similarly, a 5HT_1A_ antagonist prevented the anxiolytic, antistress, and antidepressive-like effects of acute, subchronic (7 days) (Resstel et al., 2009; Zanelati et al., 2010; Campos et al., 2012a; Twardowschy et al., 2013), or chronic (14 days) systemic administration of CBD (Campos et al., 2013b).

The molecular mechanism by which CBD facilitates 5HT_1A_ receptor activation remains unclear. Evidence suggests that it may involve allosteric modulation of this receptor, promoting 5HT_1A_ agonist-related stimulation of [35S]GTPγS binding (Russo et al., 2005; Rock et al., 2012), increase in 5-HT release and/or reuptake inhibition (Linge et al., 2016) or the indirect formation of heterodimers consisting of 5HT_1A_ and other receptors, such as CB_1_ (Mato et al., 2010).

### TRPV1 and the effects of CBD

CBD and other non-psychotomimetic phytocannabinoids can also act, at least in some cases, via the transient receptor potential vanilloid (TRPV) ion channel receptor family. CBD and cannabidivarin (CBDV) activate and desensitize TRPV1 *in vitro* (Iannotti et al., 2014). TRPV1 receptors activation contributes to the bell-shaped dose-response curve of the anxiolytic action of CBD. The lack of effects observed with high doses of CBD was prevented when the animals were treated with a TRPV1 antagonist (Campos and Guimarães, 2009). TRPV1 also seem to participate in the antihyperalgesic effects of CBD (Costa et al., 2004) as well as in the CBD effects on the sensorimotor gating disruption induced by NMDA antagonists (Long et al., 2006).

### Neuroplasticity and CBD effects in chronic stress

Several studies have addressed the effects of CBD administration in different models of stress. Among these models, chronic unpredictable stress (CUS) produces anxiety and depression-like behaviors and cognitive impairment, which are accompanied by reduced levels of neurotrophins (i.e., BDNF and others important for neuronal survival), impaired hippocampal neurogenesis and dendritic arborization, and neuroinflammatory response (microgliosis and astrogliosis; Farooq et al., 2012; Campos et al., 2013b). Chronic CBD administration counteracts the behavioral and neuroplastic consequences of CUS in adult mice by a complex interplay of different mechanisms (Campos et al., 2013b).

#### CBD and hippocampal adult neurogenesis

Adult neurogenesis is a complex process that involves division, survival (not all cells that divide will survive), migration, and differentiation of new cells (Kempermann, 2008; Suh et al., 2009; Deng et al., 2010). Although, neuronal proliferative capacity has been reported in different brain regions (Chaker et al., 2016), it is well accepted that two areas have an effective neurogenic potential under physiological conditions: the subventricular zone (SVZ), comprising the lateral walls of the lateral ventricle, and the subgranular zone (SGZ) of the dentate gyrus of the hippocampus (Kempermann et al., 2004). Both regions have a small population of neural stem/progenitor cells that originate neurons, astrocytes, and oligodendrocytes (Kempermann and Gage, 2000).

Hippocampal neurogenesis is necessary for at least some forms of learning and memory (Kempermann and Gage, 2000). In addition, decreased adult hippocampal neurogenesis has been associated with psychiatric disorders such as anxiety, schizophrenia, and mood disorders. Disturbed adult hippocampal neurogenesis may be one of the contributors to the loss of hippocampal volume reported in patients suffering from these disorders (Sheline et al., 1996). In animal models, exposure to chronic stress induces both depressive and anxiogenic-like behavior and an impairment of the dentate gyrus subgranular zone (SGZ) neurogenesis (Gould et al., 1997; Goul et al., 1998; Tanapat et al., 1998). Snyder et al. (2011) showed that neurogenesis-deficient mice presented a heightened stress-induced depressive-like behavior and impaired hypothalamus-pituitary-adrenal axis response to stress. Interestingly, classical antidepressants increase neurogenesis in a time span similar to the latency required for their therapeutic effects (Malberg et al., 2000; Manev et al., 2001). Additionally, when neurogenesis is blocked, some behavioral effects of fluoxetine and imipramine disappear (Santarelli et al., 2003; Airan et al., 2007; David et al., 2009).

Although several reports have investigated the potential use *C. sativa* derivatives in mood and anxiolytic disorders, the effects of cannabinoids on hippocampal neurogenesis was an unexplored issue until the mid-2000's. Jiang et al. (2005) observed that chronic treatment with the synthetic cannabinoid HU210 enhanced neurogenesis in rats. Wolf et al. (2010) investigated the effect of a 6 weeks treatment with a CBD-rich diet in mice and reported an increased number of cells positive for the thymidine analog bromodeoxyuridine (BrdU) in the hippocampus, reflecting increased neural progenitor cell proliferation. This facilitation of hippocampal neurogenesis seemed to depend on CB_1_ receptors since it was absent in animals lacking this cannabinoid receptor. Another report showed that repeated CBD administration prevented the reduction in neurogenesis in a murine model of Alzheimer's disease through a PPARγ-dependent mechanism (Esposito et al., 2011).

The first study to directly investigate if the behavioral effects of repeated CBD administration are mediated by its pro-neurogenic action was performed by Campos et al. (2013a). They showed that CBD reversed the anxiogenic effect and decreased neurogenesis in CUS-exposed wild-type mice. Interestingly, reforcing the pro-neurogenic effects of CBD, the anti-stress effect of CBD was not observed in transgenic GFAP/thymidine kinase mice where neurogenesis was abolished. CBD effects were also prevented by pharmacological antagonism of CB_1_ and CB_2_ receptors, suggesting that the anti-stress effects CBD depend on facilitation of hippocampal neurogenesis through a mechanism that involves increased ECB levels (Campos et al., 2013a). Interestingly, Demirakca et al. (2011) suggested that in chronic heavy Cannabis users, higher THC and lower CBD concentrations were associated with diminished hippocampal gray matter and low cognitive performance, while higher CBD concentrations in the consumed *Cannabis* samples prevented THC-induced neurotoxic effects. To explain these findings, the authors suggested that the CBD neuroprotective effects would occur through a mechanism that facilitates hippocampal neurogenesis (Demirakca et al., 2011). However, as will be discussed bellow, the role of neurogenesis in CBD effects is complex and may depend on the stress level (Schiavon et al., 2016).

#### CBD and synaptic remodeling

Synaptic plasticity could be a major target to treat mental disorders. Indeed, besides modulating neurogenesis, pre-clinical findings suggest that usual (SSRIs) and rapid-acting antidepressants, such as ketamine, may increase or restore the synaptic function impaired by chronic stress through a modulation of plastic changes. These effects may involve dendritic spines density, dendritic length and branches, neurotrophic factors (BNDF) and synaptic proteins such as synapsin, synaptophysin, post-synaptic density protein 95 (PSD95) and metabotropic glutamate receptors (mGluR), in the hippocampus and PFC (Li et al., 2010; Duman et al., 2016). These effects are mediated by several intracellular pathways that control neuronal plasticity, protection and survival, including protein kinase B (Akt), extracellular-signal regulated kinases (Erk1/2), glycogen synthase kinase 3β (GSK3β) and mammalian target of rapamycin (mTOR, Bockaert and Marin, 2015; Duman et al., 2016).

Evidence suggests that CBD can interfere with stress-induced synaptic remodeling. CBD normalized synaptophysin levels in rats submitted to a brain damage by iron overload (da Silva et al., 2014) and produced a neuritogenic effect in PC12 cells, increasing the expression of synaptophysin and synapsin I. These effects were inhibited by a TrkA antagonist (Santos et al., 2015). In addition, CBD can modulate intracellular pathways directly related to synaptic remodeling, such as Erk1/2 and Akt, in different types of cancer cell lines (McAllister et al., 2011; Solinas et al., 2013). Its precise effects in different brain regions, however, are still unclear. For example, repeated CBD administration (14 days) enhanced contextual conditioned fear responses and decreased phosphorylated forms of Erk1/2 levels in the PFC (ElBatsh et al., 2012). Since fear acquisition took place under treatment with CBD, learning/memory facilitation could also explain/contribute to this finding.

In chronically stressed mice, repeated CBD treatment also promoted dendritic remodeling and increased the expression of PSD95, Synapsin I/II, and p-GSK3β in the hippocampus of animals submitted to CUS (Fogaça, 2016).

### CBD and antidepressant effects

The first experimental evidence indicating that CBD induces antidepressant-like effects is based on its ability to attenuate autonomic and behavioral responses induced by previously inescapable stress exposure in rats (Resstel et al., 2009). These CBD effects were blocked by pre-treatment with WAY100635, a 5HT_1A_ antagonist. Following this study, our group investigated CBD effects in animals submitted to the forced swimming test (FST) (Zanelati et al., 2010) a widely used animal model predictive of antidepressant effects (Cryan et al., 2002). Systemic CBD treatment reduced immobility time in mice, as did the prototype tricyclic antidepressant imipramine (Zanelati et al., 2010). Similar results were shown in mice submitted to the FST or the tail suspension test (TST) (El-Alfya et al., 2010; Réus et al., 2011; Schiavon et al., 2016). More recently, the antidepressant-like effect of CBD was detected in the olfactory bulbectomy (Linge et al., 2016) and learned helplessness models (Pereira et al., 2016). CBD was also effective in rat strains that naturally express “depressive-like behaviors,” such as the Wistar-Kyoto (Shoval et al., 2016). Altogether, this data strengthen the possibility that CBD can induce antidepressant effects. However, the mechanisms involved in this effect have only recently started to be investigated. As discussed above, similar to the acute anxiolytic effects, the acute antidepressant effect of CBD seems to depend on facilitation of 5HT_1A_ receptor-mediated neurotransmission (Zanelati et al., 2010).

Preclinical and clinical studies indicate that forebrain 5HT_1A_ receptors are important targets of antidepressant drugs (Samuels et al., 2015; Kaufman et al., 2016). The mechanisms of this effect are still unclear, but these receptors interfere in neuroplastic events that have been associated with antidepressant action such as BDNF release and neurogenesis (Mahar et al., 2014; Serafini et al., 2014; Samuels et al., 2015; Zhang et al., 2016). However, acute or sub-chronic CBD treatment failed to change hippocampal BDNF levels (Zanelati et al., 2010; Campos et al., 2012a). Réus et al. (2011) reported similar results in three brain regions (PFC, hippocampus, and amygdala) after acute treatment with doses that induced antidepressant-like effects in rats submitted to the FST. Despite these negative results, BDNF involvement in the plastic changes induced by CBD cannot be ruled out. Methodological issues such as measurement of the whole BDNF content (not distinguishing stored and released pools), problems with the punching method, and the possibility that CBD increases BDNF release in specific subregions (Fanselow and Dong, 2010; O'Leary and Cryan, 2014; Suzuki et al., 2016) indicate that additional research on this topic is needed.

Another possibility to explain CBD-induced acute antidepressant effects would be the fast modulation of the neurochemical environment in limbic brain regions. In a recent work by Linge et al. (2016), a single CBD injection induced rapid antidepressant-like effect in the olfactory bulbectomy mouse model. This effect was associated with increased extracellular 5-HT and glutamate levels in the ventromedial prefrontal cortex (vmPFC). The 5HT_1A_-receptor antagonist WAY100635 prevented the behavioral and neurochemical effects of CBD. To explain these results, the authors suggested that CBD acute effects would be mediated by disinhibition of 5-HT and glutamatergic neurotransmission in the vmPFC through the modulation of 5HT_1A_ receptor (Linge et al., 2016). In agreement with this proposal, bilateral microinjections of CBD into the vmPFC induced antidepressant-like effect in rats submitted to the FST. This effect was blocked by pre-treatment with WAY100635 or by the CB_1_ antagonist AM251 (Sartim et al., 2016). Since the antidepressant-like effect induced by the endogenous cannabinoid anandamide was also blocked by 5HT_1A_ receptor antagonist, it was hypothesized that CBD effects on 5HT_1A_ receptors would be due to indirect modulation of local 5-HT levels via CB_1_ activation. Therefore, this data support the hypothesis that acute CBD effects could involve rapid neurochemical changes, such as in the ECB and serotonergic systems, in the vmPFC. CBD effects in other brain regions remain to be examined.

Repeated administration of CBD can prevent the impairment in neurogenesis induced by CUS (Campos et al., 2013b). At a lower dose, CBD decreased depressive-like behaviors in non-stressed Swiss mice in the TST and increased the number of Ki67, BrdU, and doublecortin-positive cells, reflecting an enhanced SGZ neurogenesis (Schiavon et al., 2016). In this study, however, a higher CBD dose, despite still displaying an antidepressant-like effect, decreased the number of positive cell markers for the neurogenesis in the hippocampus (Schiavon et al., 2016). More studies, therefore, are needed to unveil an involvement of hippocampal neurogenesis in the antidepressive-like effects of CBD. Additionally, as discussed above, the participation of rapid plastic changes such as synaptic remodeling in acute CBD effects needs to be further investigated.

Epigenetics mechanisms could also be involved in the antidepressant effects of CBD. Psychiatric disorders are thought to originate from a complex interplay between genetic predisposition and environmental factors such as stress exposure. These factors may also modulate gene expression through interference with epigenetic mechanisms (Tsankova et al., 2007; Borrelli et al., 2008). They include covalent DNA modifications (e.g., DNA methylation), post-translational modifications of histone tails (e.g., methylation, acetylation, phosphorylation, and ubiquitination), as well as non-translational gene silencing mechanisms (e.g., micro-RNAs, ribonucleic acid; Krishnan and Nestler, 2008; Kim et al., 2009). Epigenetic changes have been related to the neurobiology of various neuropsychiatric disorders, including depression (Tsankova et al., 2007; Klengel et al., 2014), and antidepressant drugs can interfere with these changes. For example, antidepressants decrease the activity of DNA methyltransferases *in vitro* (Zimmermann et al., 2012) and alter gene transcription via epigenetic mechanisms in different brain structures associated with depression (Toffoli et al., 2014). Consistent with the idea that decreasing stress-induced DNA methylation could induce antidepressant effects, administration of DNMT inhibitors (Sales et al., 2011) as well as DNMT1 knockout (Morris et al., 2016) induced antidepressant-like effects in the FST.

A recent work by Pucci et al. (2013) demonstrated that CBD could also modulate epigenetic mechanisms by reducing global DNA methylation in human keratinocytes cells. Since stress appears to increase DNA methylation, this finding raised the possibility that the antidepressant-like effects of CBD involve epigenetic mechanism, such as DNA methylation.

### CBD and antipsychotic effects

Preclinical and clinical studies indicate that CBD also has a potential therapeutic role in schizophrenia. CBD attenuates schizophrenia-related behavioral abnormalities (i.e., psychostimulant-induced hyperlocomotion, decreased sensorimotor gating, deficits in cognitive function, and decreased social interaction) in pharmacological, genetic, and neurodevelopmental animal models (Moreira and Guimarães, 2005; Gururajan et al., 2012; Long et al., 2012; Levin et al., 2014; Gomes et al., 2015a,b; Pedrazzi et al., 2015; Renard et al., 2016) with a profile similar to atypical antipsychotics (Zuardi et al., 1991, 1995; Guimarães et al., 2004). In humans, the antipsychotic properties of CBD were confirmed in a double-blind clinical trial where CBD reduced psychotic symptoms with a similar efficacy to the atypical antipsychotic amisulpride, but with significantly fewer side effects (Leweke et al., 2012). More recently, a placebo-controlled clinical trial with 88 schizophrenia patients who remained on their antipsychotic medication and were randomized to receive CBD or placebo as adjunct therapy also showed that it was consistently superior to placebo in alleviating psychotic symptoms (GW Pharmaceuticals, 2015). In this study, CBD also tended to improve negative and cognitive symptoms. Current antipsychotics show limited effectiveness in targeting these symptoms (Elvevag and Goldberg, 2000; Hanson et al., 2010).

The mechanisms by which CBD exert its antipsychotic effects are currently unknown. Leweke et al. showed that the alleviation of psychotic symptoms in patients treated with CBD was significantly associated with an increase in serum AEA levels (Leweke et al., 2012). In the same study, they confirmed that CBD inhibited FAAH activity *in vitro* (Leweke et al., 2012). Thus, by indirectly activating CB_1_ receptors via increased AEA levels, CBD could potentially modulate other neurotransmitters systems (Campos et al., 2012b). Additionally, a recent study using amphetamine sensitization model of schizophrenia suggest that its antipsychotic actions are dependent on the mTOR signaling pathway (Renard et al., 2016). However, other mechanisms such as anti-inflammatory and neuroprotective could also contribute to the beneficial effects of CBD in schizophrenia (Gomes et al., 2015b; Campos et al., 2016). The CBD antipsychotic effects may also involve parvalbumin-positive GABA neurons. These neurons are fast-spiking GABAergic interneurons, which synapse on the cell body or the axon initial segment of pyramidal neurons, promoting synchronization and temporal control of the information flow through the pyramidal neurons (Lewis et al., 2005). Parvalbumin-positive GABA neurons are selectively altered in schizophrenia patients (Lewis et al., 2005) and can account for abnormal circuit synchrony and cognitive deficits in this disorder (Gonzalez-Burgos and Lewis, 2012). Similar to patients, different rodent models of schizophrenia show deficits in the function of these cells (Penschuck et al., 2006; Gomes et al., 2015a; Canetta et al., 2016).

Recently, we observed that CBD attenuated the decreased number of parvalbumin-positive cells in the mPFC induced by repeated administration of the NMDA receptor antagonist MK-801 in mice (Gomes et al., 2015a). Parvalbumin loss induced by NMDA antagonists has been associated with increased oxidative stress (Behrens et al., 2007). The high-energy demands of parvalbumin interneurons make them particularly vulnerable to this process (Steullet et al., 2016). Impairment in antioxidant systems can lead to a diffusion of excessive reactive oxygen species outside the parvalbumin cell and induce glial activation (Morishita et al., 2015). However, glial changes could also be a consequence of high local levels of glutamate due to parvalbumin interneuron dysfunction, which leads to disinhibition of glutamate release from pyramidal neurons targeted by those interneurons (Nakazawa et al., 2012). Given that glial cells have a pivotal role in glutamate homeostasis (Bezzi et al., 1999; Shaked et al., 2005), changes in their activity may result from a compensatory mechanism in an attempt to normalize, for example, glutamate levels.

In our study, besides the changes in parvalbumin expression, the MK-801 treatment increased the expression of astrocytic and microglial cell markers in the mPFC (Gomes et al., 2015b), similar to what has been observed in *post-mortem* brain of schizophrenia patients (Radewicz et al., 2000; Catts et al., 2014). These effects were also attenuated by repeated CBD treatment (Gomes et al., 2015b) by mechanisms that are now under investigation. Suitable candidates are its antioxidant properties, activation of PPARγ or CB_1_ and/or CB_2_ receptors by the indirect increase in AEA levels (Campos et al., 2012b). These mechanisms can also be involved in the attenuation of increased glial reactivity by CBD in animal models related to other neuropathological conditions (Mecha et al., 2013; Perez et al., 2013; Schiavon et al., 2014).

### CBD and neuroprotective mechanisms

Neuroprotection constitutes an important mechanism of neuropsychiatric drugs' action to preserve structure and function of neural cells, promoting a protection against oxidative stress, iron, excitotoxicity, protein aggregation, organelles damage, and inflammation (Kaur and Ling, 2008; Filipović et al., 2017). An imbalance in these processes are found in animal models of depression, anxiety, stroke and neurodegenerative diseases such as Alzheimer's, Huntington's, Parkinson's, and multiple sclerosis (Kaur and Ling, 2008; Filipović et al., 2017).

Antioxidants compounds act against oxidative stress, a condition characterized by an exacerbation of reactive oxygen/nitrogen species (ROS/RNS) production and, consequently, peroxidation of polyunsaturated fatty acids, DNA oxidation, and nitration/carbonylation of proteins, leading to cell damage or death (Pisoschi and Pop, 2015). One of the first studies relating CBD to neuroprotection showed that it acts as an antioxidant, preventing NMDA- and kainate receptor-mediated neurotoxicity and hydroperoxide-induced oxidative damage in rat cortical neuron culture, through a mechanism independent of cannabinoid receptors (Hampson et al., 1998). In these assays, CBD demonstrated a superior neuroprotective activity than other known antioxidants such as alpha-tocopherol and ascorbate (Hampson et al., 1998). Other studies have shown that neuroprotective effects of CBD are associated with its antioxidant properties (Table 2). CBD decreases the neuronal damage promoted by β-amyloid protein deposit (Iuvone et al., 2004; Esposito et al., 2006, 2011; Sagredo et al., 2007; Harvey et al., 2012; Janefjord et al., 2014; Scuderi et al., 2014) and attenuates the depletion of tyrosine hydroxylase, dopamine, and GABA levels by modulating the expression of the inducible isoform of nitric oxide synthase and reducing the production of ROS-generating NADPH oxidases (Esposito et al., 2006, 2007; Garcia-Arencibia et al., 2007; Sagredo et al., 2007, 2011; Pan et al., 2009). These effects seem to occur, in part, by activation of PPARγ receptors (Esposito et al., 2011; Scuderi et al., 2014) and ubiquitination of amyloid precursor protein (Scuderi et al., 2014). CBD protective responses were also accompanied by an increase in cell survival through inhibition of ROS/RNS production, a decrease in malondialdehyde and caspase-3 levels, inhibition of DNA fragmentation (Iuvone et al., 2004), and reduces de activity of NF-κB (Kozela et al., 2010). In addition, CBD exerts antioxidant activities against toxicity and/or oxidative stress produced by H_2_O_2_, tertbutyl hydroperoxide, and amphetamine (Valvassori et al., 2011; Harvey et al., 2012; Mecha et al., 2012). Moreover, CBD pre-treatment attenuates high-glucose-induced mitochondrial superoxide generation and NF-κB activation, along with the expression of the adhesion molecules ICAM-1 and VCAM-1 (Rajesh et al., 2007).

**Table 2:** **CBD and neuroprotective mechanisms**.

**Main effect of CBD**	**Model**	**CBD Dose/concentration range**	**Route of administration**	**Species/Strain**	**Possible mechanism of action**	**References**
Prevents NMDA receptor-induced excitoxicity	E17 cortical neurons culture	EC50 = 3.7 μM	*In vitro*	Wistar rat	Effect independent of cannabinoid receptors.	Hampson et al., 1998
↓Phosphorylated form of p38/MAP kinase, ↓Caspase 3 levels, and NFκ-b activation	β amyloid-induced neurotoxicity in PC12 cells	10 μM	*In vitro*	PC12 cells	Antioxidant	Esposito et al., 2006
Prevented gliosis, neuronal death and ↑ hippocampal neurogenesis	Genetic model of Alzheimer's Disease	10 mg/kg	15 days	C57BL6 mice	PPARγ	Esposito et al., 2011
↓Aβ cell viability and ↓LPS (conditioned media) induced microglia activation	β amyloid -induced neuronal toxicity in neuroblastoma cells. LPS-induced microglial-activation	10 μM	*In vitro*	Neuroblastoma (SH-SY5Y) cells/Microglial (BV-2) cells	Not determinated	Janefjord et al., 2014
Improved cell viability	Amyloid β -induced toxicity and tert-butyl hydroperoxide-induced oxidative stress	0.01–10 μM	15 min pre-incubation before Aβ or sAβ addition/ 24-h incubation for oxidative stress analysis	PC12 and Neuroblastoma (SH-SYS5) cells	Not determinated	Harvey et al., 2012
↓ Amyloid- β production	Amyloid β -induced neurotoxicity	100 nM	24 h	SHSY5Y (APP+) neurons	PPARγ	Scuderi et al., 2014
Reversed 3-nitropropionic acid—induced ↓ GABA contents, ↓ substance P, ↓ neuronal-specific enolase and superoxide dismutase(SOD)-2	(10 mg/kg) 3-nitropropionic acid-induced) striatal lesions	5 mg/kg	5 days, i.p.	Sprague-Dawley rats	Independent of CB_1_, TRPV_1_ and A_2A_ receptors	Sagredo et al., 2007
↓ Levels of IL-1beta, GFAP and iNOS	Amyloid β -induced neurotoxicity	10 mg/kg	i.p.	C57BL6 mice	Not determinated	Esposito et al., 2007
Reduced dopamine depletion and ↑mRNA levels of SOD in the substantia nigra	6-hydroxydopamine toxicity	3 mg/kg	14 days, i.p.	Sprague-Dawley rats	Antioxidant	Garcia-Arencibia et al., 2007
↓Cell death	H_2_O_2_-inducedoxidative stress in Oligodendrocyte progenitor cells	1 μM	*In vitro*	Oligodendrocyte progenitor cells	Not determinated	Mecha et al., 2012
↓of carbonyl groups and prevents the decrease in BDNF expression	Amphetamine-induced oxidative stress	60 mg/kg	2 weeks, i.p.	Wistar rats	Not determinated	Valvassori et al., 2011
↓ NFκ-B, ↓ ICAM-1 and VACAM-1	High glucose-induced mithocondrial superoxide generation	4 μM	*In vitro*	Human coronary artery endothelial cells	Independent from CB1 and CB2 receptors	Rajesh et al., 2007
Prevented Aβ-induced cognitive deficits, ↓ microglia activation, ↓ IL-6 mRNA expression Inhibited NO generation and ATP-induced intracellular Ca2+ levels	Rat primary cortical cultures, N13 and BV-2 microglial cells Morris water maze	10–1,000 nM	*In vitro* 3 weeks: first week treated daily; second and third weeks treated 3 times/week, i.p.	Rat primary cortical cultures, N13 and BV-2 microglial cells C57BL6 mice	Some of the *in vitro* effects were mediated by A_2A_, CB_1_, and CB_2_ receptors	Martín-Moreno et al., 2011
Blocked LPS-induced STAT1 activation	LPS-induced BV-2 activation	10 μM	*In vitro*	BV-2 microglial cells	Not determinated	Kozela et al., 2010
↓Apoptosis; ↓Excitotoxicty and neuroinflamation	Newborn hypoxic-ischemic brain damage	0.1–1,000 μM	*Ex vivo*	Brain slices from C57BL6 mice	CB_2_ and A_2A_ receptors	Castillo et al., 2010
Protects against the reduction in tyrosine hydroxylase activity	6-hydroxydopamine-induced toxicity in the striatum and substantia nigra	3 mg/kg	14 days, i.p.	Sprague-Dawley rats	Not determinated	Lastres-Becker et al., 2005
↑ Viable neurons and ↓ excitoxicity, oxidative stress, and inflammation	Newborn hypoxic-ischemic brain damage (HI)	1 mg/kg	30 min after HI, i.p.	Newborn pigs	CB_2_ and 5HT_1A_ receptors	Pazos et al., 2013
Improve of cognition and motor activity. Restores BDNF levels	Encephalopathy (bile duct ligation)	5 mg/kg	28 days, i.p.	C57BL6 mice	5HT_1A_	Magen et al., 2010
Improvments od liver function, normalizes 5-HT levels, and improves brain pathology	Encephalopathy (thioacetamide)	5 mg/kg	Single dose	C57BL6 mice	5HT-dependent mechanism	Avraham et al., 2011
Faciltates autophagic flux and decrease oxidative stress	Pilocarpine-Induced Seizure	100 ng	Intracerebroventricular	Wistar rats	Induction of autophagy pathway	Hosseinzadeh et al., 2016
Suppresses the transcription proinflammatory genes	MOG35-55-specific T cell in the presence of spleen-derived antigen presenting cells	5 μM	*In vitro*	MOG35-55- and APCs isolated from spleens of C57BL6	Not determinated	Kozela et al., 2016
Attenuates TNF-α production and ↓ adenosine transport	murine microglia and RAW264.7 macrophages LPS-treated mice	500 nM or 1 mg/kg	*In vitro*	Murine microglia	A_2A_ adenosine receptor	Carrier et al., 2006
			*In vivo* (1 h before LPS injection, i.p.)	RAW264.7 macrophages C57BL6 mice		
Improves motor deficits in the chronic phase; ↓ microglial activation and Il-beta and TNF-α production	Viral model of multiple sclerosis	5 mg/kg	7 days, i.p	SJL/J mice	A_2A_ adenosine receptor	Carrier et al., 2006
Normalizes synaptophyisin and caspase 3 expression	Brain damage induced by iron overload during neonatal period	Not informed	14 day, i.p.	Wistar rats	Not determinated	da Silva et al., 2014
Prevented MPP-induced toxicity and induces neurite growth	MPP-induced toxicity in PC12 cells and SH-SY5Y	1 μM	*In vitro*	PC12 and SH-SY5Y cells	TRKA	Santos et al., 2015
Prevents cognitive and anxiogenic effects, ↓ TNF-α and IL-6 ↑ BDNF levels	Murine model of cerebral Malaria	30 mg/kg	10 days, i.p.	C57BL6 mice	Not determinated	Campos et al., 2015

*i.p., intra peritoneal; ↓, decreases; ↑, increases*.

In models based on lipopolysaccharide (LPS) media activation, CBD increased microglial viability and migration as well as inhibited nitric oxide generation and STAT1 activation induced by LPS (Kozela et al., 2010; Martín-Moreno et al., 2011; Janefjord et al., 2014). Some of these effects were mediated by adenosine A_2A_, CB_1_, and/or CB_2_ receptors. In addition, CBD demonstrated neuroprotection in forebrain slices from newborn mice that underwent hypoxic-ischemic brain damage, reducing glutamate, IL-6 levels, and the expression of TNFalpha, COX-2, and iNOS via activation of CB_2_ and adenosine A_2A_ receptors (Castillo et al., 2010). In accordance with these *in vitro* results, CBD was also neuroprotective *in vivo* after acute or chronic treatment in animal models of neurodegenerative diseases (Lastres-Becker et al., 2005; Alzheimer's and Parkinson's, Esposito et al., 2007, 2011; Garcia-Arencibia et al., 2007; Sagredo et al., 2007), ischemia (Pazos et al., 2013) and encephalopathy (Magen et al., 2010; Avraham et al., 2011). In a model of β-amyloid hippocampal inoculation, CBD decreased IL-1beta, GFAP, and iNOS levels (Esposito et al., 2007). In models of Parkinson's disease, CBD reversed the reduction of tyrosine hydroxylase activity and the dopamine depletion in the substantia nigra and striatum after 6-hydroxydopamine microinjection (Lastres-Becker et al., 2005; Garcia-Arencibia et al., 2007) and upregulated superoxide dismutase (SOD) mRNA levels in the substantia nigra (Garcia-Arencibia et al., 2007). In this same direction, CBD also reversed 3-nitropropionic acid-induced reductions in GABA contents and mRNA levels for substance P, neuronal specific enolase and superoxide dismutase 2, effects that were independent of CB_1_, TRPV1, and A_2A_ receptors (Sagredo et al., 2007).

Moreover, in hypoxic-ischemic animals, CBD prevented the decrease in the number of viable neurons and the increase in excitotoxicity, oxidative stress, and inflammation through mechanisms involving CB2 and 5HT_1A_ receptors (Pazos et al., 2013). In the middle cerebral artery occlusion, a method used to evaluate ischemia-reperfusion injury, CBD suppressed the decrease in cerebral blood flow after reperfusion, inhibited myeloperoxidase (MPO) activity in neutrophils, and reduced the number of MPO immunopositive cells. In addition, in animal models of encephalopathy, CBD improved cognition, motor activity, and restored 5-HT and BDNF levels via 5HT_1A_ receptor activation (Magen et al., 2010; Avraham et al., 2011).

Besides these proposed mechanisms, neuroprotection can also be promoted by an enhancement in the autophagic function. Autophagy, more specifically macroautophagy, is a lysosomal degradation pathway essential to recycle damaged organelles and promote cell survival, protecting the cell malfunction or death under stress conditions (Jia and Le, 2015). In fact, some SSRIs and mood stabilizers such as lithium increase autophagy in the brain (Heiseke et al., 2009; Gassen et al., 2014; Jia and Le, 2015). Although there are few studies relating CBD to autophagy in neuropsychiatric disorders, CBD can modulate this process (Koay et al., 2014; Yang et al., 2014; Hosseinzadeh et al., 2016). Specifically in the brain, CBD produced anticonvulsant effects concomitant to an activation of hippocampal autophagy pathway in the chronic phase of pilocarpine-induced seizure (Hosseinzadeh et al., 2016). In a genetic model of tauopathy, used to evaluate frontotemporal dementia, parkinsonism, and motor neuron disease, 1-month daily injections of Sativex®, a 1:1 mixture combination of CBD and THC, increased the ratio of reduced/oxidized glutathione and promoted autophagy in the brain (Casarejos et al., 2013). In this same study, Sativex® attenuated the abnormal behaviors and reduced free radicals produced during the metabolism of dopamine, iNOS levels and deposition of tau in the hippocampus and cortex (Casarejos et al., 2013). In this context, recent findings from our group suggest that chronic CBD treatment increase autophagy in animals submitted to CUS, as revealed by its impact in phosphorylated form of mTOR, Beclin-1 and LC3, signaling proteins involved in autophagy induction (Fogaça, 2016).

The CBD neuroprotective effects could also involve neuroinflammatory mechanisms. CBD administration counteracts the deleterious consequences of neuroinflammation reducing adaptive immune cell Th2 phenotype and microglial/macrophage innate immunity (Kozela et al., 2016).

CBD inhibits adenosine transporter activity in a microglial cell line, thus resulting in increased A_2A_ receptor signaling, and attenuation of TNF-α cytokine production (Carrier et al., 2006). Likewise, adenosine 2A receptor signaling is involved in the protective effects of CBD in the Theiler virus model of multiple sclerosis preventing both leukocyte and microglial-mediated actions (Mecha et al., 2013). On the other hand, CBD protects oligodendrocyte progenitor cells in CB_1_, CB_2_, PPARγ, and TRPV1 independent manner (Mecha et al., 2012).

## Conclusions

In addition to CBD-only actions, considerable interest is also derived from its ability to modulate the psychoactive effects of THC. CBD tempers the detrimental (cognitive impairment, psychosis) consequences of THC (Curran et al., 2016), while preserving its beneficial actions. For example, administration of a THC: CBD mixture 1:1 (the similar composition to Sativex) in the APPxPS1 exhibits a better therapeutic profile than each cannabis component alone (Aso et al., 2015). Similarly THC:CBD combination is neuroprotective actions in toxin-induced striatal neurodegeneration (Valdeolivas et al., 2012). In a recent double-blind crossover pilot clinical trial, Sativex administration in Huntington's disease patients was shown to be safe and well tolerated, while no significant effects or biomarker changes could be evidenced (López-Sendón et al., 2016). Similarly to CBD, other non-psychotomimetic phytocannabinoids exert significant neuroplasticity adaptations of therapeutic interest by this mechanism. Cannabigerol (CBG) a non-CB_1_/CB_2_ receptor acting cannabinoid shares with CBD its ability to activate PPARγ and has been evaluated in preclinical models of neurodegeneration (Valdeolivas et al., 2015). New molecules derived from the CBG structure have been tested in multiple sclerosis and Huntington's disease models (toxin- and mutant huntingtin-induced striatal neurodegeneration, Granja et al., 2012; Díaz-Alonso et al., 2016). Characterization of the neuroprotective actions of VCE3.2 compound indicates that it acts as a PPARγ modulator without the secondary actions of full PPARγ agonists, thus highlighting the translational implications offered by non-psychotomimetic cannabinoids.

The pre-clinical and clinical studies available so far indicate that CBD has a good safety record (Bergamaschi et al., 2011b). CBD or other non-psychotomimetic phytocannabinoids effects in brain development, however, have not yet been extensively investigated. This lack of studies is a significant point given that endogenous cannabinoid system regulates important steps of the nervous system development (Diaz-Alonso et al., 2012). The deleterious consequences in brain development and psychiatric implications in the adulthood induced by psychoactive THC exposure have been widely studied (Tortoriello et al., 2014; Alpár et al., 2015; de Salas-Quiroga et al., 2015). Recent evidence indicates that while CBD in the adult brain is safe and lacks undesired side effects, in the differentiating neurons it may increase their sensitivity to future oxidative insults (Schönhofen et al., 2015). Clearly, research on CBD safety during brain development is essential.

Even with this unanswered question, however, CBD ability to reduce inflammation-associated neurodegeneration and its antioxidant properties, lack of psychoactivity and a broad range of potentially beneficial effects indicates that this drug could be a useful new approach to treat several neuropsychiatric disorders. Actually, new CBD-derived molecules aimed at improving the efficacy and/or potency of natural phytocannabinoids have been recently developed (Haj et al., 2015; Breuer et al., 2016).

**Figure 1:**
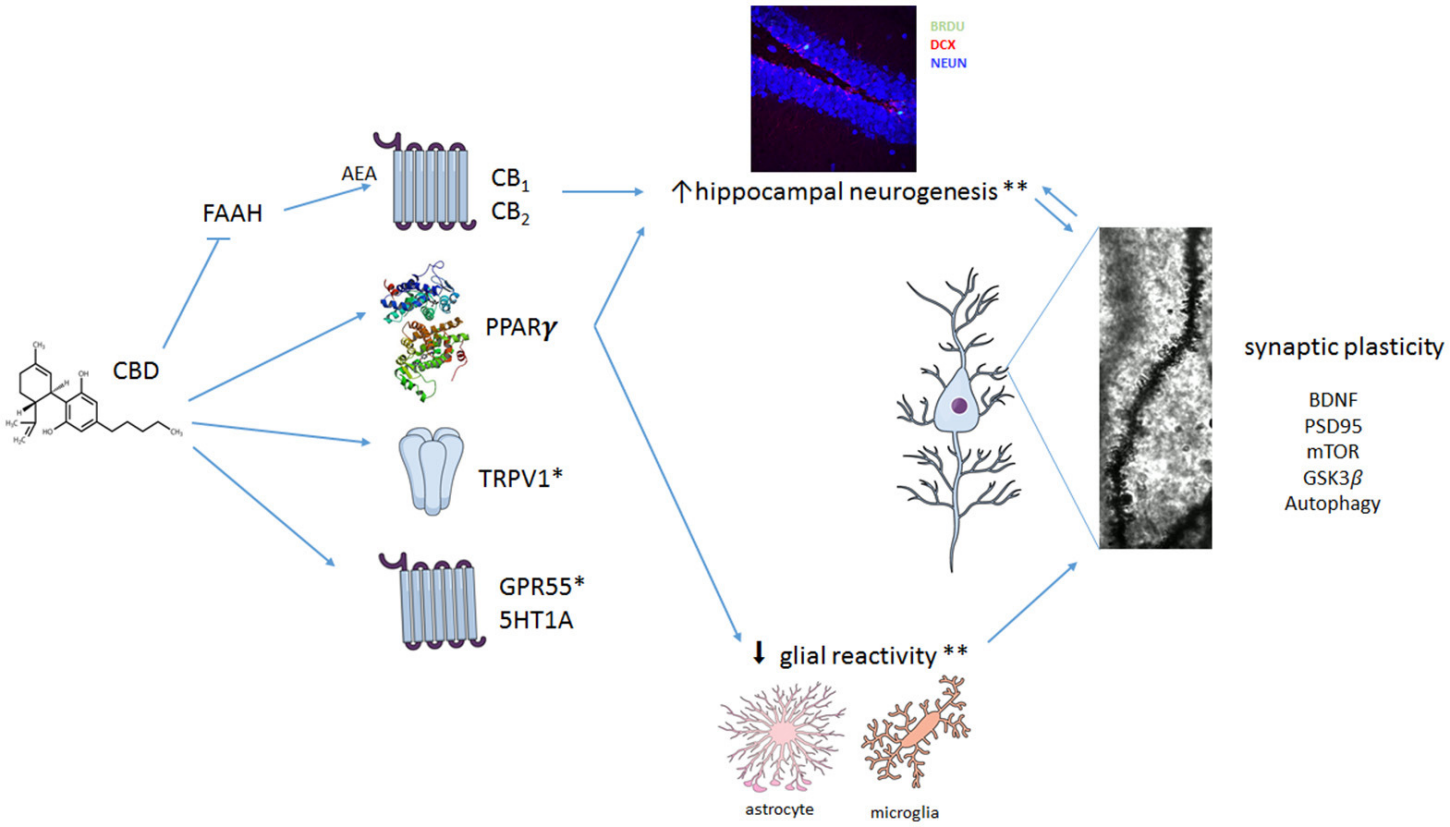
**Multiple mechanisms proposed to explain CBD effect in neuropsychiatric disorders**. CBD seems to interact with numerous different targets. It can act as a positive modulator of 5HT_1A_-mediated neurotransmission or as an agonist at TRPV1 and PPARγ receptors. In addition, CBD can facilitate anandamide (AEA)-mediated neurotransmission (by inhibiting FAAH) and induce antioxidant actions. CBD also promotes a complex set of changes in crucial intra-cellular pathways such as mTOR, autophagy and GSK3β, resulting in neuroprotection, decreased proinflammatory responses and facilitation of neuroplastic events. Taken together, these mechanisms would lead to an overall beneficial effect of CBD in neuropsychiatric disorders. ^*^Little is known about the contribution of TRPV1 and 5HT1A for the effects of CBD on glial reactivity and on adult hippocampal neurogenesis. ^**^CBD effects in reducing glial cell reactictivity and preventing stress-induced or amyloid-β-induced decreased adult hippocampal neurogenesis seem to depend on activation of CB1/CB2 (indirectly) and PPARγ. Because CB1 and CB2 are expressed in both neural precursor cells (NPCs) and glial cells, CBD effects on adult hippocampal neurogenesis could be a result of its actions on NPCs and/or attenuation of glial reactivity. BDNF, Brain derived neurotrophic factor; PSD95, postsynaptic density protein 95; mTOR, mechanistic target of rapamycin; GSK3β, Glycogen synthase kinase 3-beta.

As discussed above, different mechanisms appear to be involved in CBD effects (Figure 1). The evidence available so far indicates that, in addition to its better described acute mechanisms (such as interaction with 5HT_1A_-mediated neurotransmission, TRPV1 receptors, and inhibition of anandamide metabolism), plastic changes take place over time and contribute to CBD-induced behavioral effects in response to chronic treatment. Even if the number of studies investigating these chronic effects is still sparse, it is clear that no single mechanism will explain the remarkable pharmacological profile of CBD. In this way, it joins a club of multi-target drugs that includes, for example, clozapine. These drugs challenge our familiar concept that acting in a single pharmacological target is always desirable (Imming et al., 2006) and agree with the observation made by Mencher and Wang (2005) that, sometimes, promiscuity could be a virtue.

## Author contributions

AC, MF, FS, SJ, AJS, FVG, ABS, NR, and IG participated in the writing process of the first draft of the manuscript. AC design the Figure 1. FSG, AC, and IG revised the final version of the manuscript.

### Conflict of interest statement

FG is co-inventor of the patent “Fluorinated CBD compounds, compositions and uses thereof.” Pub. No.: WO/2014/108899. International Application No.: PCT/IL2014/050023. The other authors declare that the research was conducted in the absence of any commercial or financial relationships that could be construed as a potential conflict of interest.
